# Diuretic response to Ringer's solution is normal shortly after awakening from general anaesthesia: a retrospective kinetic analysis

**DOI:** 10.1016/j.bjao.2022.100013

**Published:** 2022-05-21

**Authors:** Robert G. Hahn, Joel Olsson

**Affiliations:** 1 Research Unit, Södertälje Hospital, Södertälje, Sweden; 2 Karolinska Institutet at Danderyds Hospital (KIDS), Stockholm, Sweden; 3 Department of Anaesthesia, Sundsvalls sjukhus, Sundsvall, Sweden

**Keywords:** acetated Ringer's solution, blood, fluid balance, hemodilution, infusion, laparoscopic cholecystectomy, pharmacokinetics, surgery

## Abstract

**Background:**

The elimination of Ringer's solution is severely depressed during general anaesthesia, but the degree to which this continues postoperatively is poorly established.

**Methods:**

An intravenous infusion of Ringer's acetate solution 20 ml kg^−1^ was administered over 60 min in 12 patients undergoing laparoscopic cholecystectomy. Population kinetic analysis was performed based on repeated measurements of blood haemoglobin concentration and urinary excretion over 240 min regardless of when the operations were finished. The analysis contrasted the periods before and after awakening from general anaesthesia and compared them with data from 18 volunteers who received the same fluid at the same rate.

**Results:**

Patients were monitored for approximately 2 h after awakening from general anaesthesia. The rate constant for redistribution of fluid from the extravascular space to the plasma (*k*_21_) and the rate constant for urinary excretion (*k*_10_) were significantly higher postoperatively than during the surgical period. Computer simulations indicated that urinary excretion after surgery was almost restored to the rate found in the volunteers. In contrast, the redistribution of fluid from the extravascular space to the plasma, which was almost nil during the surgery, showed only limited recovery during the postoperative phase, and was only approximately 10% of the flow rate found in the volunteers. The combination of nearly normalised urinary excretion and lack of adequate return of distributed fluid to the plasma promoted postoperative hypovolaemia.

**Conclusion:**

The kinetic analysis indicates that plasma volume support should be given during the first 2 h after laparoscopic cholecystectomy.

Intravenous infusion of crystalloid fluid with a nearly iso-osmotic content of electrolytes remains the mainstay of plasma volume support during surgery.^1^^,^^2^ Older studies have reported fewer postoperative complications when providing Ringer's solution at 3–5 ml kg^−1^ h^−1^ rather than at 10–12 ml kg^−1^ h^−1^,^3^^,^^4^ whereas the more recent and larger REstrictive versus LibEral Fluid Therapy in major abdominbal surgery (RELIEF) trial found the opposite effect.^5^ Urinary excretion is known to be strongly inhibited during anaesthesia and surgery (–90%),^6^, ^7^, ^8^ which makes a ‘restrictive’ fluid regimen logical as a poor diuretic response to plasma volume expansion easily results in fluid overload.

The duration of poor renal excretion of infused fluid after surgery is unclear. Holte and co-workers^9^ reported accelerated excretion of Ringer's solution 4 h after laparoscopic cholecystectomy. Myles and colleagues^10^ still suggest that the postoperative fluid strategy should be restrictive owing to the frequently encountered weight increase of 3–4 kg after surgery. However, recent data show that the postoperative fluid balance might correlate poorly with weight gain.^11^

The aim of the present study was to contrast the turnover of fluid during the first 2 h after laparoscopic cholecystectomy against the fluid kinetics measured during the preceding period of anaesthesia and surgery. The results were compared with infusion experiments performed in conscious volunteers. The hypothesis was that normal fluid kinetics would only be partially restored in the immediate postoperative period because of the residual effects of the anaesthetics.

## Methods

This report is a retrospective analysis based on data derived from three published prospective studies where data were collected in the same way. In the first study, plasma dilution was measured as a surrogate for plasma volume expansion when Ringer's acetate solution was administered to 12 patients during elective laparoscopic cholecystectomy.^12^ The duration of the study was 240 min, and data collection continued into the postoperative period. The results were compared with data derived from 18 healthy conscious volunteers who received the same fluid at the same rate (Table 1).^13^^,^^14^

**Table 1:** Studies from which the experiments were derived. ∗Matched with date from Olsson and colleagues^12^ based on rate of infusion. ^†^Nine control subjects received Ringer.

Study	Total no. of experiments	Experiments in this study	Number of subjects
Olsson and colleagues^12^	12	12	12
Hahn and colleagues^13^	30	10∗	6
Sjöstrand and colleagues^14^	30^†^	8	8

The protocols for the original studies were approved by the Ethics Committee of Huddinge University Hospital (Dnr. 56-00, 168/91, and 276/96), and each subject provided informed written consent to participate. Eleven of the 12 patients had been included in the first study, the female volunteers in the second, and the male volunteers in the third.

### Experimental procedure

The patients were allowed to have a light morning breakfast before the study. No premedication was given. With the subject in the supine position, a cannula was placed in the antecubital vein of each arm, one for blood sampling and the other for fluid infusion. General anaesthesia was induced using propofol, rocuronium, and remifentanil, and maintained with remifentanil and sevoflurane as described previously.^12^ The pneumoperitoneum was maintained with the patient in the reverse Trendelenburg position. No intravenous fluid was given during induction of anaesthesia.

When surgery started, an intravenous infusion of acetated Ringer's solution 20 ml kg^−1^ was given at a constant rate over 60 min via an infusion pump. Ringer's solution has the following ionic content: Na^+^ 130, K^+^ 4, Ca^2+^ 2, Mg^2+^ 1, Cl^−^ 110, and acetate 30 mmol L^−1^. The osmolality is approximately 270 mOsm kg^−1^. Monitoring included pulse oximetry and electrocardiography. Noninvasive arterial pressure was measured in the arm not used for fluid infusion by an automatic device. The excreted urine was measured every 15 min via an indwelling catheter, which had been inserted into the bladder after the induction of anaesthesia but before the infusion started. No other fluid was given during the experiment.

The volunteers were given 25 ml kg^−1^ of the same fluid over 45 min (*n*=14) or 80 min (*n*=4). Selection was made from a larger cohort to achieve the same average infusion rate as in the patients. Urine was voided spontaneously halfway through and at the end of each experiment.

Venous blood samples, 3 ml each, were collected every 5 min during the first 90 min and every 10 min during the subsequent 150 min. All samples were analysed for blood haemoglobin (Hb) concentration at the same hospital's certified clinical chemistry laboratory, with a coefficient of variation (CV) of 1–2%.

### Basic kinetic model

A two-volume kinetic model with three rate constants (*k*_12_, *k*_21_, and *k*_10_) and one scaling factor between dilution and volume (*V*_c_, central volume) was fitted to the dependent variables (frequently measured plasma dilution and total urinary excretion).^15^

**Fig 1:**
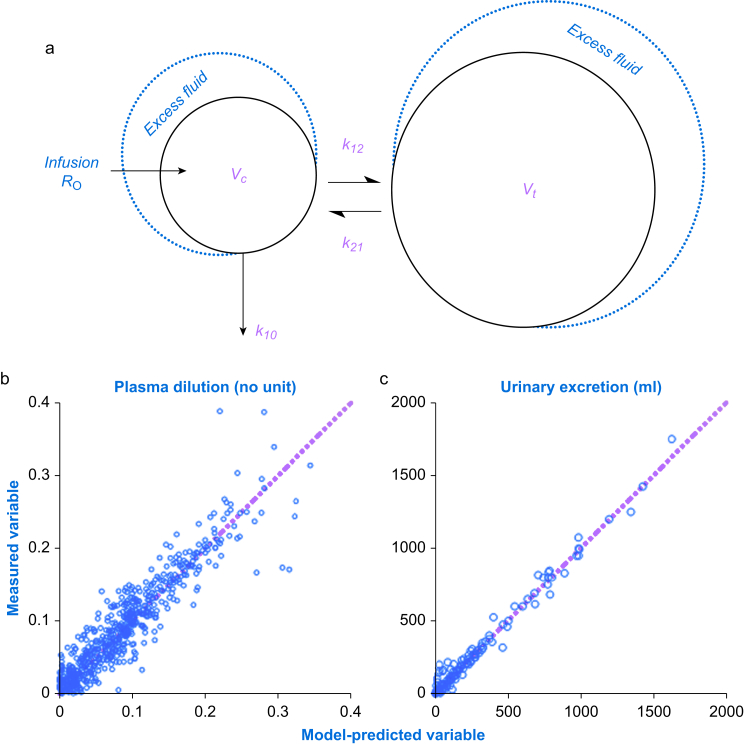
(a) Schematic drawing of the kinetic model. (b) Comparison between the measured and model-predicted urinary excretion. (c) Comparison between the measured and model-predicted plasma dilution.

Fluid is infused at the rate *R*_o_ into the plasma (*V*_c_) from which distribution occurs to (*k*_12_) and is redistributed from (*k*_21_) an extravascular space (*V*_t_). Elimination from *V*_c_ occurs by urinary excretion (*k*_10_). All flow rates are proportional by one of these rate constants (*k*_12_, *k*_21_, and *k*_10_) to the volume expansion of the respective body fluid space (Fig. 1a).

The differential equations are:

d*v*_c_/d*t*=*R*_o_–*k*_10_(*v*_c_–*V*_c_)–*k*_12_(*v*_c_ – *V*_c_)+*k*_21_(*v*_t_–*V*_t_)

d*v*_t_/d*t*=*k*_12_(*v*_c_–*V*_c_)–*k*_21_(*v*_t_–*V*_t_)

d*U*/d*t*=*k*_10_(*v*_c_–*V*_c_)

Baseline volumes are given in capital letters (*V*_c_ and *V*_t_) whereas expanded volumes are shown in lower-case letters (*v*_c_ and *v*_t_), and *U* is the urinary excretion. Hence, the volume expansion of the central fluid space is given by (*v*_c_–*V*_c_).

The Hb-derived fractional plasma dilution used to indicate the volume expansion of *V*_c_ resulting from the infusion was given by:

(*v*_c_–*V*_c_)/*V*_c_=[(Hb/hb)–1]/(1–baseline haematocrit)

Each dilution was slightly corrected to account for blood sampling.^16^

### Covariate analysis

The fluid volume kinetics during general anaesthesia and surgery are described by the parameters in the basic model (*V*_c_, *k*_12_, *k*_21_, and *k*_10_) to which covariates were added to evaluate possible changes in kinetics during the postoperative period, and any difference from the volunteers. The criterion for accepting ‘postoperative’ or ‘volunteer’ as covariate in the model required that its inclusion reduced the –2 LL (log likelihood) for the model by >6.6 points (*P*<0.01).^15^ Moreover, the estimate of the covariate needed to have a 95% confidence interval (CI) that did not include 0. How inclusion of covariates mathematically changed the parameter estimates is explained in the Appendix.

### Calculations

The kinetic model was simultaneously fitted to all measurements of plasma dilution and urinary excretion (dependent variables) in the 30 infusion experiments using the Phoenix 8.3.4 software for non-linear mixed effects (Pharsight, St. Louis, MO, USA) with the First-Order Conditional Estimation Extended Least Squares (FOCE ELS) as a search routine. The sandwich method was used as the variance estimator, as it is robust for covariance misspecification. The programme derived *k*_10_ by averaging the urinary excretion over time regardless of how frequently urine was collected.

The parameters ultimately reported represented a ‘Full block model’, in which the inter-individual variability of the fixed effects and their covariates (theta:s) are supplemented by the variances and the covariance variability of the random effects (eta:s). A full block model generates the most precise simulations.

Goodness-of-fit and performance of the final model were evaluated by predictive checks, residual plots, and plots of the conditional weighted residuals (CWRES).^17^

Simulations and plots were performed by inserting the optimal parameter values derived by the analysis into the same kinetic model programmed in MATLAB R2018b (MathWorks, Inc., Natick, MA, USA).

Data showing a normal distribution were reported as the mean and standard deviation (sd), and changes were evaluated using paired *t* test. Kinetic parameters were reported as the best estimate and 95% CI according to the output from the Phoenix software. The significance levels for inclusion of the covariates were taken from the Phoenix software.

## Results

### Subjects and experiments

Thirty subjects were studied: 12 were patients (female/male ratio, 11:1) who underwent laparoscopic cholecystectomy. They were 43 (11) yr old and weighed 75 (11) kg.

The 18 control subjects were volunteers (female/male ratio, 10:8). They were 29 (7) yr old and weighed 70 (13) kg.

The average infused volumes were 1.51 (0.21) *vs* 1.32 (0.35) L, which were administered at rates of 25 (4) and 26 (7) ml min^−1^, respectively.

Patients were monitored for 90 (33) min during ongoing surgery and for an additional 140 (33) min after general anaesthesia had been reversed.

### Kinetic analysis

Kinetic analysis was performed on all 812 data points on a single occasion. Data on urinary excretion were available on 131 occasions (average, 4.4 per experiment).

### Simulations

**Fig 2:**
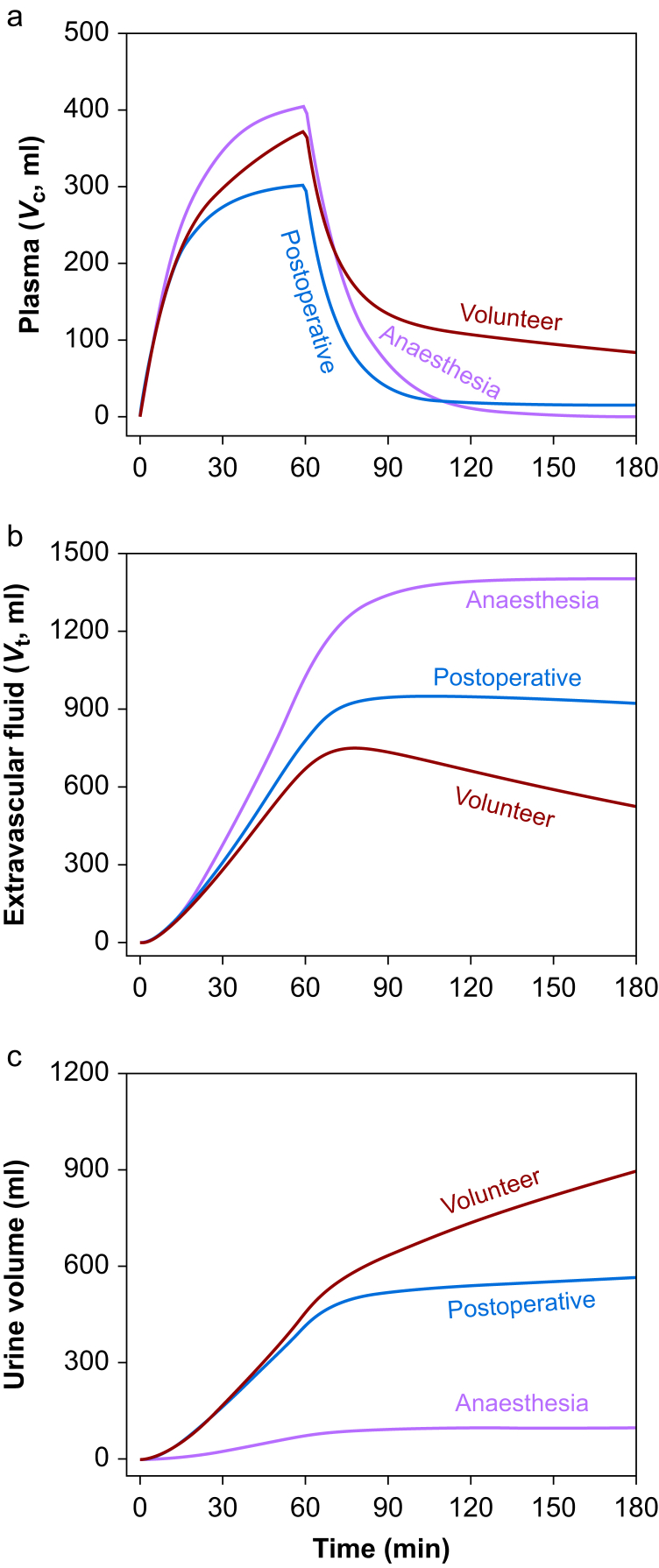
Simulation of the distribution of 1.5 L Ringer's acetate solution between (a) plasma, *V*_c_; (b) extravascular space, *V*_t_; and (c) urine, when administered at a constant rate over 60 min. Three sets of parameters were derived from the data shown in Table 1, and each one of them was then used to simulate an entire volume–time curve.

Three different sets of parameters were derived from the data shown in Table 1. The first one represented the kinetic situation during ongoing general anaesthesia and the second the kinetic situation during the postoperative period in the same patients. The third set of parameters represented the fluid kinetics in the conscious volunteers who had received the same infusion fluid at approximately the same rate as the patients during surgery. These three sets of parameters were used to create the plots shown in Figure 2, which predict the distribution and elimination of a simulated infusion of Ringer 1.5 L over 60 min.

Compared with the kinetic parameters obtained during general anaesthesia, the parameters representing the postoperative period were characterised by slightly smaller plasma volume expansion in response to the simulated fluid load (Fig. 2a). Moreover, preference for extravascular deposition of the fluid was attenuated (Fig. 2b), and the diuretic response to fluid loading was almost restored (Fig. 2c).

**Fig 3:**
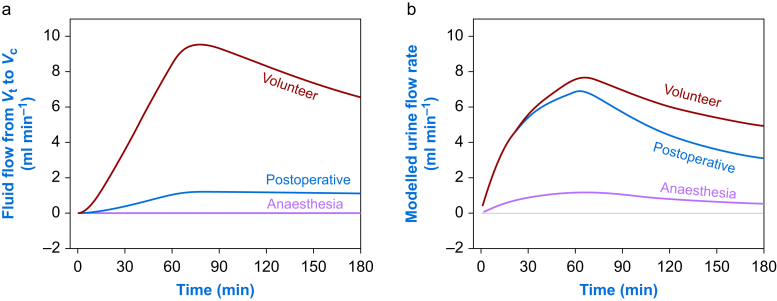
(a) Simulation of the redistributive flow of fluid from extravascular space (*V*_t_) to plasma (*V*_c_) and (b) urine flow rate when 1.5 L Ringer's acetate solution is administered at a constant rate over 60 min. The best estimates from Table 1 were used. The flow rate in subplot (a) was given by *k*_21_ (*v*_t_–*V*_t_) and in subplot (b) by *k*_10_ (*v*_c_–*V*_c_).

Simulation of the *flow rates* showed that the redistribution of fluid from the extravascular space to the plasma was still retarded postoperatively (Fig. 3a), whereas the diuresis agreed well with the urine flow rate found in the volunteers (Fig. 3b).

## Discussion

### Key results

The diuretic response to infused Ringer's solution was much poorer during surgery than in conscious volunteers, but it was almost restored in the early postoperative period after laparoscopic cholecystectomy. The covariance analysis showed a 7-fold increase in the rate constant for urinary excretion (*k*_10_) during the first postoperative 2 h compared with anaesthesia and ongoing surgery.

In contrast, the rate constant for the redistribution of fluid from the extravascular space to the plasma (*k*_21_) was not normalised after the surgery. This rate constant was almost nil during the surgery, but not even the strong postoperative (hundred-fold) increase in *k*_21_ was sufficient to restore the return flow to the plasma.

Our simulation of a fluid infusion based on postoperative data illustrates that the normalised diuresis combined with lack of adequate return flow of distributed fluid promotes hypovolaemia.

### Interpretation

Differences in kinetic parameter estimates are difficult to interpret without the use of simulation plots. Here, we even simulated a postoperative infusion, although no such infusion was given. The plot based on the postoperative data indicates that plasma volume support should be given to patients awakening from general anaesthesia because they are vulnerable to hypovolaemia at that time.

As shown in Figure 2a, the maximum plasma volume expansion induced by Ringer's solution was 20–25% poorer when the simulation was based on postoperative data as compared with the simulation based on the kinetic parameters derived from the period of anaesthesia and surgery. However, the crucial period began when the infused fluid had been distributed 30 min after the infusion was turned off. Adequate flow of fluid returning from the extravascular space then maintained the plasma volume in volunteers, but not in postoperative patients. Figure 3a shows that the return flow was very slow, which means that the plasma did not receive volume support from already distributed fluid despite accelerated diuresis. The combination of these findings soon created a situation where practically all the infused fluid volume that remained in the body was in the extravascular space.

### Implications

A restrictive fluid therapy is a rational strategy during surgery because of the strong inhibition of the diuretic response, which increases the plasma volume expansion induced by crystalloid fluid. The hormones involved in the ‘stress response’ to trauma would promote retention of fluid and salt also in the postoperative period, but the degree of the extension probably depends on the magnitude of the surgery.^18^ However, the scant literature on this topic does not uniformly support the restrictive approach after surgery.

The RELIEF trial found plasma creatinine elevation to be more frequent in the fluid restricted group, in which the strategy extended into the postoperative period.^5^ The investigators administered 0.8 ml kg^−1^ h^−1^ after the surgery, which is the minimum requirement of fluid in adults.^19^ The same authors recommend intravenous titration of fluid in case a patient is unable to drink,^20^ but a restrictive approach is still recommended postoperatively.^10^ In contrast, a blinded randomised trial by Vermeulen and co-workers^21^ compared restrictive with standard fluid therapy after surgery and found a higher incidence of various complications, even serious ones, in the restrictive group. However, these studies involved different types of operations that were usually of long duration (average 3 h).

A study in patients undergoing laparoscopic cholecystectomy, where the operating time is usually 1 h, reported improved organ function and recovery when using a liberal fluid regimen (40 ml kg^−1^) as compared with a restrictive fluid programme (15 ml kg^−1^).^22^ In another study by Holte and colleagues,^9^ the diuretic response to crystalloid fluid was stronger 4 h after laparoscopic cholecystectomy than before the surgery.

The Enhanced Recovery After Surgery (ERAS) programme encourages the earliest possible return to oral intake of fluid,^18^ which can be 5–6 h after laparoscopic cholecystectomy. Henriques and Correia^23^ found no reason to provide any intravenous fluid while awaiting oral intake to begin. Most other studies suggest that intravenous fluid should be given, although this remains to be confirmed.

Henriques and Correia^23^ did not administer any fluid after the operation, just as in the present study. However, our kinetic data give reason to continue providing fluid postoperatively because the plasma volume expansion induced by fluid during the surgery soon disappears. Hypovolaemia might even ensue because of our demonstrated combination of slow redistribution and restored diuretic response to volume expansion. The downside of continuing to infuse fluid is that it will aggravate peripheral oedema (expansion of *V*_t_), as slow redistribution implies that the extravascular space is not yet able to adequately clear excess fluid.

### Kinetic analysis

The kinetic approach to the analysis and simulation of the distribution of infused fluid has close similarities to drug pharmacokinetics. The model is intended to mimic physiological body fluid spaces, where the plasma corresponds to *V*_c_ and the interstitial fluid volume to *V*_t_. The flow given by *k*_12_ is intended to represent the capillary leakage and *k*_21_ primarily the lymphatic return. At steady state, the relationship between *k*_12_ and *k*_21_ mimics the relationship between *V*_c_ and *V*_t_. The fluid kinetic model seems to represent these physiological correlates reasonably well on a whole-body level, provided that the Starling forces are not abruptly changed during an experiment.^15^^,^^24^

During surgery, a second elimination compartment might exist alongside *V*_t_ that does not communicate with the plasma during a normal experimental period of 3–4 h.^25^ Fluid accumulation in a slowly equilibrating peripheral compartment could be the reason why *k*_21_ was almost zero during the surgery. However, the current data set was not sufficiently large to model this second compartment.

Likewise, the arterial pressures probably affect *k*_10_,^7^ but our data could not capture this covariance because nine parameters were already estimated from the cohort of only 30 patients. Plots of the arterial pressures and the measured urine flow rate over time are shown in the original publication of the patient group.^12^ The arterial pressures and urinary volumes are also available in Supplementary Digital File 1.xls of the present report.

### Limitations and strengths

The poor return of fluid from the extravascular space might be peculiar to the laparoscopic positioning of the patient, as data from thyroid surgery, where the patient rests in the supine position, show a normal *k*_21_.^15^^,^^16^ Therefore, the reported results should not be uncritically extrapolated to other surgical operations. The study used Ringer's acetate, whereas lactated Ringer's is more widely used internationally. These fluids show quite similar distributions and eliminations in volunteers.^26^ Only the acetated solution is marketed in Scandinavia owing to better results during lactic acidosis in hypokinetic animals.^27^ The present report uses data from previous work,^12^, ^13^, ^14^ but refinement of the analytical method using population kinetics has opened up the possibility of disclosing more precisely what happens to the fluid in the body when a patient awakens from anaesthesia.

Strengths of the study include the fact that the kinetic analysis had a good ability to re-create the measured data, as shown in Figure 1b. The validity of the analysis is further increased by the fact that the surgical patients were their own controls. All measurements were performed in a standardised fashion by the same research team. We believe that the results are generalisable to all patients who undergo laparoscopic cholecystectomy.

## Conclusions

A kinetic analysis of the fluid kinetics during the first 2 h after laparoscopic cholecystectomy shows that the diuretic response to fluid, which was poor during the surgery, was soon restored to almost the same level as that in volunteers. However, the return flow of distributed fluid to the plasma remained very poor, which promoted peripheral oedema and slight hypovolaemia. Consequently, plasma volume support of modest intensity seems to be warranted in the early postoperative phase after laparoscopic cholecystectomy.

## Authors' contributions

Patient recruitment and data collection: JO, RGH.

Literature search: JO.

Study design, data analysis, and manuscript preparation: RGH.

## Acknowledgements

The authors are grateful to the collaborators who helped collect the data from the original studies for creation of this database.

## Declarations of interest

RGH has received a research grant from 10.13039/501100016387Grifols for studies of 20% albumin and is Member of Baxter's IV Fluid Therapy management Advisory Board. JO declares that he has no conflicts of interest.

## Figures and Tables

**Table 2:** Population kinetic parameters for infusion of Ringer's solution during 12 laparoscopic cholecystectomies. The covariates represent modifications of the parameters representing the first 2 h postoperatively in the same patients and in 18 volunteers receiving Ringer's at the same infusion rate. Covariance for *k*_21_ and *k*_10_ was evaluated for ‘postoperative’ and ‘volunteers’ in the same run. CI, confidence interval; CV%, coefficient of variation (inter-individual); LL, log likelihood for the model during development. Decrease by >6.6 points=*P*<0.01; tv, typical value for the group.

Kinetic parameter	Covariate	Best estimate	95% CI	CV%	–2 LL
tv*V*_c_ (L)		2.73	2.34 to 3.13	7.3	
tv*k*_10_ (10^−3^ min^−1^)		3.90	2.18 to 5.62	22.4	
tv*k*_12_ (10^−3^ min^−1^)		56.1	4.16 to 7.07	13.2	
tv*k*_21_ (10^−6^ min^−1^)		6.15	2.43 to 9.88	30.8	–1371
*k*_21_	Postoperative	5.42	4.38 to 6.45	9.8	
*k*_21_	Volunteer	7.65	6.83 to 8.46	5.4	–1402
*k*_10_	Postoperative	2.00	1.05 to 2.96	24.3	
*k*_10_	Volunteer	1.99	1.46 to 2.51	13.4	–1449
*V*_c_	Conscious	–0.24	–0.35 to –0.11	–26.2	–1460
Full block	All of the above	7.65	6.83 to 8.46	5.4	–1475

## Appendix

The following text explains how the parameter estimate representing the diuretic response to central volume expansion (*k*_10_) changed after anaesthesia and surgery had been finished. For this purpose, the exponential covariate model was used.^15^ In the spreadsheet used for the kinetic analysis (Supplementary File 1.xls), all measurements performed during anaesthesia (abbreviated anaesth) were marked by 0 and all data obtained postoperatively in the same patient by 1. The value *k*_10_ during each period of the experiment is then given by:

*k*_10_=*k*_10__anaesth_[e^cov anaesth=0, postop=1^],

where e=2.718 and Cov is the computer-estimated value of the covariate. During anaesthesia and surgery, the covariate effect is absent because e^0^=1. The value of *k*_10_ during anaesthesia is then equal to its typical value (tv*k*_10_) shown in Table 2 to be 0.0039 min^−1^, which corresponds to a half-life of 3 h (given by ln 2/*k*_10_).

The covariate value of 2.00 comes into play during the postoperative period. Here, *k*_10_ is given by 0.0039∗(2.718 ˆ 2.00), which is 0.0288 min^−1^ and corresponds to an intravascular half-life of only 24 min. Hence, there was a dramatic improvement in diuretic response to fluid after surgery. This half-life would normally be prolonged by fluid redistributed from *V*_t_ at a rate determined by *k*_21_ but, as shown below, this fluid source was poorly available after the surgery.

The covariate analysis also included volunteer experiments. Therefore, each time point in all subjects represented either the Anaesthesia, Postoperative, or Volunteer setting and was marked as 0, 1, or 2. This combined covariate was analysed in one single run but the output reported two exponents. The covariate values for *k*_10_ were virtually identical for the postoperative period and in volunteers (2.00 *vs* 1.99), which shows that *k*_10_ was normal postoperatively. This was not the case for *k*_21_, which is the rate constant describing redistribution of infused fluid. The equations expressing *k*_21_ in these settings are:

*k*_21_=*k*_21__anaesth_×[e^cov anaesth=0, postop=1^]

*k*_21_=*k*_21__anaesth_×[e^cov anaesth=0, volunteer=1^]

The value *k*_21_ for the postoperative period, based on the data from Table 2, becomes 6.15×10^−6^ min^−1^×(2.718 ˆ 5.42)=0.0015 min^−1^, which corresponds to a half-life of 7.7 h. The corresponding value of *k*_21_ for volunteers is 6.15×10^−6^ min^−1^×(2.7187.65)=0.0136 min^−1^, which corresponds to a half-life of only 50 min.
