# Supplementary material for: Diuretic response to Ringer's solution is normal shortly after awakening from general anaesthesia: a retrospective kinetic analysis
Source: BJA Open. 2022 May 21;2:100013. doi: 10.1016/j.bjao.2022.100013 (PMC10430821; doi:10.1016/j.bjao.2022.100013)
Supplement: Multimedia component 2 [file mmc2.pdf]

Phoenix output, selected files

1. Theta
2. Omega
3. Eta
4. Program code
5. Theta covariance
6. Omega standard errors
7. Eta covariance
8. Eta eta
9. Post hoc theta
10. Theta correlation

1. Theta

	Parameter	Estimate	Units	Stderr	CV%	2.5% CI	97.5% CI
	tvV	2737.4927		200.50595	7.3244379	2343.9733	3131.0121
	tvKe	0.0039021913		0.0008756513	22.439989	0.00218361	0.0056207725
	tvK12	0.056109141		0.0074098608	13.206156	0.041566311	0.07065197
	tvK21	6.1546509E-06		1.8964258E-06	30.812891	2.4326649E-06	9.8766368E-06
	dK21dWakeup1	5.4170706		0.52839121	9.7541874	4.3800331	6.454108
	dK21dWakeup2	7.6519551		0.41394743	5.409695	6.8395287	8.4643816
	dKedWakeup1	2.0036694		0.48722835	24.316804	1.0474194	2.9599193
	dKedWakeup2	1.9852933		0.26527344	13.361927	1.4646592	2.5059274
	dVdVakenNa1	-0.23592517		0.061882096	-26.229544	-0.35737695	-0.11447339
	stdev0	0.03129778		0.0034068494	10.885275	0.024611389	0.037984172
	stdev1	53.519323		9.9167122	18.529218	34.056467	72.982179

2. Omega

io	Label	nKe	nK12	nK21	nV
	Omega				
	nKe	1.4987005			
	nK12	0.52104012	0.42737619		
	nK21	0.08196366	0.33890797	1.1213376	
	nV	-0.26716206	-0.20327635	-0.25653146	0.18255546
	Correlation				
	nKe	1			
	nK12	0.65104136	1		
	nK21	0.063226003	0.48956282	1	
	nV	-0.51076361	-0.72775346	-0.56698951	1
	Shrinkage	0.060657545	0.16894974	0.15033844	0.17995051

3. Eta

Case_no	nKe	nK12	nK21	nV	nKe_shrinkage	nK12_shrinkage	nK21_shrinkage	nV_shrinkage
1	-0.72088	0.551692	2.31808	-0.265711	0.033628	0.108881	0.01105	0.210979
2	1.79286	0.787835	0.0744079	-0.260557	0.066186	0.231935	0.148333	0.437053
3	0.902971	0.473099	-0.00335958	-0.336516	0.068917	0.176829	0.311453	0.329707
4	0.366489	-0.0618704	-1.26501	0.187641	0.118907	0.125188	0.648089	0.208037
5	0.221473	-1.39862	-2.21267	0.94291	0.010918	0.02154	0.568065	0.04209
6	-0.0333932	0.510371	0.679078	-0.000819455	0.07159	0.177471	0.139082	0.33691
7	-0.700817	0.0265699	0.00975845	-0.0637151	0.062398	0.059823	0.200704	0.112478
8	-0.181658	-0.374957	-1.1973	0.174503	0.032033	0.045702	0.504723	0.076729
9	-0.720273	-0.709384	-0.502386	0.171856	0.022473	0.033578	0.326831	0.048809
10	-2.67496	-1.35305	1.28611	0.328285	0.136482	0.032407	0.050791	0.027848
11	-1.65472	-0.418899	0.355924	-0.149652	0.056339	0.017204	0.047952	0.027047
12	1.48567	0.183271	-0.0973619	-0.0259053	0.039631	0.152532	0.446789	0.283781
21	0.96322	0.0174196	-0.171074	-0.125962	0.053687	0.430055	0.11394	0.443938
22	-0.253565	-0.0209123	-0.0847448	-0.0879973	0.029963	0.299847	0.066941	0.266582
24	-0.000259541	-0.0334306	0.120153	-0.77493	0.02694	0.286367	0.05624	0.223822
26	-0.89651	-0.0295778	-0.136928	-0.0659739	0.037934	0.327887	0.083451	0.29389
27	1.80465	0.799463	0.60354	-0.424223	0.066548	0.48812	0.100208	0.509957
28	0.189929	0.341326	-0.384419	0.0946857	0.030872	0.237423	0.102144	0.28026
29	0.231705	-0.220058	-0.259522	-0.0845371	0.020084	0.22345	0.108396	0.184379
30	-0.1481	-0.014427	0.879355	-0.475198	0.020742	0.336419	0.056965	0.163663
31	-1.40418	0.0366455	0.919712	0.113802	0.031874	0.423426	0.073876	0.204698
32	-0.719472	-0.145407	0.419452	0.119342	0.023712	0.314714	0.075847	0.189256
150	0.568668	-0.0994982	-0.906964	0.0767016	0.057433	0.428075	0.440945	0.396266
151	2.33506	0.938543	1.4156	-0.550869	0.165326	0.747922	0.62018	0.79275
152	-0.440137	0.211399	-0.583374	0.343788	0.078673	0.406954	0.26146	0.418776
153	-0.364454	0.218382	-0.555747	0.327701	0.076377	0.410743	0.256667	0.420251
154	1.98444	0.830125	1.43137	-0.546353	0.12336	0.694027	0.435711	0.651955
155	0.410735	-0.0194183	0.188632	0.0145767	0.047537	0.441045	0.142131	0.326747
156	0.0933059	0.0308188	-0.349781	0.200574	0.055918	0.411925	0.217901	0.364628
157	-1.9824	-0.55379	-0.63832	0.608385	0.116685	0.34943	0.291404	0.223537

Cases 1-12 are cholecystectomies, Cases 13-30 are volunteers

4. Program code

```
test(){
		deriv(A1 = - (A1 * Ke)- (A1 * K12- A2 * K21))
		urinecpt(A0 = (A1 * Ke))
		deriv(A2 = (A1 * K12- A2 * K21))
		C = A1 / V
		dosepoint(A1, idosevar = A1Dose, infdosevar = A1InfDose, infratevar = A1InfRate)
		error(CEps = 0.0312989449053771)
		observe(CObs = C + CEps)
		error(A0Eps = 53.5176796609099)
		observe(A0Obs = A0 + A0Eps)
		stparm(V = tvV * exp(dVdVakenNa1*(VakenNa==1)) * exp(nV))
		stparm(Ke = tvKe * exp(dKedWakeup1*(Wakeup==1)) * exp(dKedWakeup2*(Wakeup==2)) *
exp(nKe))
		stparm(K12 = tvK12 * exp(nK12))
		stparm(K21 = tvK21 * exp(dK21dWakeup1*(Wakeup==1)) *
exp(dK21dWakeup2*(Wakeup==2)) * exp(nK21))
		fcovariate(Wakeup())
		fcovariate(VakenNa())
		fixef(tvV = c(, 2724.20277648683, ))
		fixef(tvKe = c(, 0.00392916929720172, ))
		fixef(tvK12 = c(, 0.056214079033135, ))
		fixef(tvK21 = c(, 6.0098459346898E-06, ))
		fixef(dK21dWakeup1(enable=c(0)) = c(, 5.43763579542067, ))
		fixef(dK21dWakeup2(enable=c(0)) = c(, 7.67048619874927, ))
		fixef(dKedWakeup1(enable=c(1)) = c(, 1.99832568003605, ))
		fixef(dKedWakeup2(enable=c(1)) = c(, 1.97946560192064, ))
		fixef(dVdVakenNa1(enable=c(2)) = c(, -0.3, ))
		ranef(block(nKe, nK12, nK21, nV) = c(1.5051022, 0.52671886, 0.43299065, 0.083678585,
0.35237771, 1.1428697, -0.27074577, -0.20757175, -0.26506324, 0.18521494))
}
```

Wakeup = postoperative; Vaken = conscious.

5. Theta covariance

tvV	tvKe	tvK12	tvK21	dK21dWakeup1	dK21dWakeup2	dKedWakeup1	dKedWakeup2	dVdVakenNa1	stdev0	stdev1
40202.636										
-0.057005256	7.667652E-07									
-1.1183182	3.9931884E-06	5.4906037E-05								
0.00021420511	-1.1309317E-09	-9.4201654E-09	3.596431E-12							
-42.214604	0.00015658775	0.0019442772	-6.5128551E-07	0.27919727						
-44.572416	0.00020995519	0.0019562173	-7.1020638E-07	0.147581	0.17135248					
-9.9218384	-5.8066529E-05	-0.00061854558	1.245469E-07	-0.16108067	-0.035130864	0.23739146				
-2.4135346	-0.00011122767	-0.0002956192	7.1542491E-08	0.007341459	-0.0047974901	-0.012677179	0.070369996			
3.6075059	-1.5659126E-05	-0.00018980665	6.0745418E-08	-0.018037063	-0.013998611	0.01285141	-0.0013491836	0.0038293938		
-0.071066488	-1.8660506E-07	3.1516974E-06	-4.8407315E-10	-7.2877933E-05	0.00010489132	-9.1701314E-05	3.8540207E-06	-0.0001073701	1.1606623E-05	
866.88904	0.0021796788	-0.025842747	6.6028116E-06	-1.0438036	-1.2032381	-1.0262809	-0.39529352	0.11505396	-0.011705221	98.34118

6. Omega standard errors

	Scenario	Label	nKe	nK12	nK21	nV
1		nKe	0.47534361			
2		nK12	0.267591	0.12236693		
3		nK21	0.23252555	0.18252818	0.40849076	
4		nV	0.15612368	0.071755129	0.10906174	0.063400469

7. Eta covariance

Case_no	Wakeup	VakenNa	nKe	nK12	nK21	nV
1	0	0	-0.72087963	0.5516922	2.3180847	-0.2657106
2	0	0	1.7928597	0.78783491	0.074407935	-0.26055652
3	0	0	0.90297111	0.4730995	-0.0033595769	-0.33651578
4	0	0	0.36648921	-0.061870363	-1.2650135	0.18764079
5	0	0	0.22147294	-1.3986164	-2.2126709	0.94290972
6	0	0	-0.033393223	0.51037085	0.67907774	-0.00081945514
7	0	0	-0.70081654	0.026569897	0.0097584512	-0.063715085
8	0	0	-0.18165848	-0.37495706	-1.1973007	0.17450273
9	0	0	-0.72027254	-0.70938404	-0.50238637	0.17185648
10	0	0	-2.6749624	-1.3530507	1.2861099	0.32828518
11	0	0	-1.6547199	-0.41889877	0.35592443	-0.14965205
12	0	0	1.485668	0.183271	-0.097361885	-0.025905295
21	2	1	0.9632197	0.017419576	-0.17107351	-0.12596179
22	2	1	-0.25356474	-0.020912263	-0.084744794	-0.087997338
24	2	1	-0.00025954099	-0.033430625	0.12015308	-0.77492996
26	2	1	-0.8965095	-0.029577811	-0.13692799	-0.065973938
27	2	1	1.8046511	0.79946305	0.60354018	-0.42422289
28	2	1	0.189929	0.34132637	-0.38441923	0.094685693
29	2	1	0.23170523	-0.22005772	-0.25952193	-0.084537105
30	2	1	-0.14810043	-0.014427028	0.87935453	-0.47519778
31	2	1	-1.4041791	0.036645502	0.91971197	0.11380203
32	2	1	-0.71947177	-0.14540687	0.41945171	0.11934189
150	2	1	0.56866834	-0.099498178	-0.90696368	0.07670161
151	2	1	2.3350629	0.93854335	1.4156017	-0.55086852
152	2	1	-0.44013719	0.21139874	-0.58337447	0.34378796
153	2	1	-0.36445391	0.21838176	-0.55574722	0.32770119
154	2	1	1.9844396	0.83012463	1.4313669	-0.54635289
155	2	1	0.41073544	-0.019418318	0.1886321	0.014576747
156	2	1	0.093305937	0.030818795	-0.34978064	0.2005737
157	2	1	-1.9824045	-0.55379031	-0.63832045	0.60838541

8. Eta eta

Case_no	Eta1Name	Eta2Name	Eta1	Eta2	NPEta1	NPEta2
1	nKe	nK12	-0.72087963	0.5516922		
1	nKe	nK21	-0.72087963	2.3180847		
1	nKe	nV	-0.72087963	-0.2657106		
1	nK12	nKe	0.5516922	-0.72087963		
1	nK12	nK21	0.5516922	2.3180847		
1	nK12	nV	0.5516922	-0.2657106		
1	nK21	nKe	2.3180847	-0.72087963		
1	nK21	nK12	2.3180847	0.5516922		
1	nK21	nV	2.3180847	-0.2657106		
1	nV	nKe	-0.2657106	-0.72087963		
1	nV	nK12	-0.2657106	0.5516922		
1	nV	nK21	-0.2657106	2.3180847		
2	nKe	nK12	1.7928597	0.78783491		
2	nKe	nK21	1.7928597	0.074407935		
2	nKe	nV	1.7928597	-0.26055652		
2	nK12	nKe	0.78783491	1.7928597		
2	nK12	nK21	0.78783491	0.074407935		
2	nK12	nV	0.78783491	-0.26055652		
2	nK21	nKe	0.074407935	1.7928597		
2	nK21	nK12	0.074407935	0.78783491		
2	nK21	nV	0.074407935	-0.26055652		
2	nV	nKe	-0.26055652	1.7928597		
2	nV	nK12	-0.26055652	0.78783491		
2	nV	nK21	-0.26055652	0.074407935		
3	nKe	nK12	0.90297111	0.4730995		
3	nKe	nK21	0.90297111	-0.0033595769		
3	nKe	nV	0.90297111	-0.33651578		
3	nK12	nKe	0.4730995	0.90297111		
3	nK12	nK21	0.4730995	-0.0033595769		
3	nK12	nV	0.4730995	-0.33651578		
3	nK21	nKe	-0.0033595769	0.90297111		
3	nK21	nK12	-0.0033595769	0.4730995		

9. Post hoc theta

id	time	V	Ke	K12	K21
1	0	2098.7288	0.0018977304	0.097416045	6.2507888E-05
2	0	2109.5737	0.023438922	0.1233633	6.630074E-06
3	0	1955.2668	0.0096264003	0.090052986	6.1340085E-06
4	0	3302.5114	0.0056295385	0.052742858	1.7370608E-06
5	0	7028.3512	0.0048695971	0.0138555	6.7336829E-07
6	0	2735.2504	0.0037740362	0.093472716	1.2137329E-05
7	0	2568.5135	0.0019361892	0.057619937	6.2150047E-06
8	0	3259.4066	0.003253983	0.038564867	1.8587558E-06
9	0	3250.7928	0.0018988828	0.02760273	3.7240868E-06
10	0	3801.241	0.00026889773	0.014501442	2.2271725E-05
11	0	2357.0018	0.00074588665	0.036906953	8.7857621E-06
12	0	2667.4878	0.017239552	0.067394926	5.5836693E-06
21	0	1906.2834	0.074444305	0.057095101	0.010917144
22	0	1980.0457	0.022048991	0.054947955	0.011901484
24	0	996.19455	0.028405194	0.054264385	0.014607878
26	0	2024.1367	0.01159208	0.054473858	0.011296353
27	0	1414.6673	0.17268734	0.12480616	0.023687514
28	0	2376.9138	0.034355447	0.078935029	0.008819707
29	0	1986.909	0.035821089	0.045026041	0.009993011
30	0	1344.3619	0.024501421	0.055305464	0.031210744
31	0	2422.7888	0.0069772331	0.058203427	0.032496092
32	0	2436.248	0.013837189	0.048515914	0.019704747
150	0	2334.5493	0.050174148	0.050795134	0.0052301722
151	0	1246.3867	0.29350492	0.14342928	0.053357236
152	0	3049.2791	0.018296218	0.069317505	0.0072285135
153	0	3000.6185	0.019734683	0.069803244	0.0074310016
154	0	1252.0277	0.20670055	0.12869218	0.05420509
155	0	2193.929	0.04284406	0.055030106	0.015643257
156	0	2642.4089	0.031191247	0.057865279	0.009130562
157	0	3972.9252	0.0039134803	0.03224969	0.0068420502

10. Theta correlation

tvV	tvKe	tvK12	tvK21	dK21dWakeup1	dK21dWakeup2	dKedWakeup1	dKedWakeup2	dVdVakenNa1	stdev0	stdev1
1										
-0.32468067	1									
-0.75271069	0.61542985	1								
0.56333495	-0.68103482	-0.67036698	1							
-0.39845553	0.33843166	0.49658379	-0.64995002	1						
-0.53702402	0.57922889	0.63776672	-0.90469774	0.67472944	1					
-0.10156225	-0.13610125	-0.1713283	0.13479212	-0.62568435	-0.17418513	1				
-0.045376657	-0.47883721	-0.15039342	0.14221141	0.052376085	-0.043689304	-0.098083584	1			
0.29074668	-0.28898234	-0.41393901	0.5176219	-0.55162662	-0.54648058	0.42623902	-0.082188733	1		
-0.10403624	-0.062551723	0.12484798	-0.074924212	-0.040484383	0.074377472	-0.05524463	0.0042644923	-0.50929028	1	
0.43598198	0.25101148	-0.35169073	0.35109556	-0.19920283	-0.29311543	-0.21240562	-0.15026513	0.187486	-0.34646469	1
